# Association between age at menarche and the risk of myopia at the age of 15 in Chongqing, China: a cross-sectional study

**DOI:** 10.3389/fmed.2025.1590463

**Published:** 2025-07-15

**Authors:** Jing Zhang, Ruili Li, Yong Zhang, Tian OuYang, Dan Ao, LiYan Gong, Li He, Xiaoya Qi, Xiyuan Zhou

**Affiliations:** ^1^Department of Health Management Center, The First Affiliated Hospital of Chongqing Medical University, Chongqing, China; ^2^Department of Special Medical Service Centre, The First Affiliated Hospital of Chongqing Medical University, Chongqing, China; ^3^School of Public Health, Chongqing Medical University, Chongqing, China; ^4^Health Medicine Center, The Second Affiliated Hospital of Chongqing Medical University, Chongqing, China; ^5^Department of Ophthalmology, The Second Affiliated Hospital of Chongqing Medical University, Chongqing, China

**Keywords:** myopia, menarche age, adolescent, Chinese girls, puberty

## Abstract

**Introduction:**

Menarche is one of the important indicators of girls’ physical, nutritional, and reproductive health. This study aims to identify the relationship between the age at menarche and the risk of myopia at the age of 15.

**Methods:**

Girls aged 15 were recruited when they visited the hospital for physical examination required for enrollment in the high school. Eligible participants underwent anthropometric measurements and an ocular examination, and they completed a questionnaire to gather personal information, including age at menarche. Ocular indexes were compared across early, normal, and late menarche groups, and multivariate regression was performed to analyze the relationships between age at menarche and myopia.

**Results:**

Among the 376 participants around the age of 15, 115 girls were classified into the early menarche group, 185 into the normal menarche group, and 76 into the late menarche group. The prevalence of myopia was 95.7, 87.6, and 86.6% for the right eye (*p* = 0.048) and 89.6, 77.8, and 68.4% (*p* = 0.001) for the left eye in the early, middle, and late menarche groups, respectively. Age at menarche was negatively and significantly associated with myopia, particularly with moderate to severe myopia in 15-year-old girls (*p* = 0.039 for the right eye, *p* = 0.001 for the left eye).

**Discussion:**

Age at menarche, as a marker of the onset of puberty, was identified as an independent risk factor of myopia in teenage girls, particularly for moderate to severe myopia. This implies that accelerated physical development during puberty may contribute to the onset and progression of myopia in the context of modern lifestyles in urban China. Additional interventions should be considered for this subgroup of children.

## Introduction

Myopia has become a significant global public health concern (1) with its rapidly increasing prevalence (2), irreversible complications (3), and heavy economic burden (4). In addition, the high prevalence of myopia was found to exist in East Asia, with the highest prevalence in China (5).

Menarche is one of the indicators of the onset of puberty in girls. It is believed to be associated with diseases in adults, such as reproductive cancers (6), metabolic syndrome (7), and cardiovascular diseases (8). Some studies have also explored the relationship between menarche and myopia. Studies based on the Korean National Health and Nutrition Examination Survey (KNHANES) (9) and the US National Health and Nutrition Examination Survey (NHANES) (10) have shown a negative association between age at menarche and myopia prevalence in adult female individuals. Teenage girls in post-menarche stage had a higher prevalence of myopia than those still in pre-menarche stage across all age groups from 10 to 15 years, according to data from the Chinese National Survey on Students’ Constitute and Health CNSSCH (1995–2014) (11). However, studies based on the CNSSCH did not account for the actual age at menarche within each age group when categorizing girls into pre-or post-menarche stages. In contrast, a cohort study that investigated the markers of puberty and myopia among children aged 6–14 years in Singapore found that early menarche (defined as age below the 50th percentile of the study cohort), or the age at menarche, was not associated with the age of myopia onset (12). However, this cohort study followed girls only until they reached grade 8 or age 14, which may not have been sufficient to detect the effect of menarche.

In addition to differences in the study design, there are variations in lifestyle across time periods and regions, which may have contributed to the differences in the findings of the studies mentioned above. Over the past decade, the average ages for the onset of myopia and menarche have decreased among Chinese adolescents, possibly due to significant changes in lifestyle (13). Therefore, it is necessary to confirm the relationship between menarche as a marker of puberty and myopia in adolescents within the context of current lifestyles.

Hence, we conducted a cross-sectional survey of female junior high school graduates in urban Chongqing, aiming to provide evidence for understanding the mechanisms underlying myopic development and to inform strategies for myopia prevention among adolescents.

## Methods

### Study participants

The participants were female junior high school graduates from urban Chongqing, approximately 15 years old, who visited the hospital for a physical examination required for senior high school enrollment. The Ethics Committee of Chongqing Medical University provided ethical approval for the study (No. CAF52704054B). The purposes and contents of the study were explained to all participants and their parents or legal guardians, and written informed consent was obtained from all participants.

Students who did not have menarche, who refused to participate, or who had pre-existing eye conditions such as strabismus, amblyopia, ocular inflammation, ocular trauma, corneal disease, congenital cataract, or choroid or retinal disorders were excluded from the study. Students with high astigmatism (astigmatism greater than three diopters or anisometropia greater than three diopters), high hyperopia (hyperopia greater than three diopters), or who wore contact lenses or orthokeratology lenses during the ocular examination were also excluded from this study.

### Ocular examinations

Ocular examinations included visual acuity testing, slit-lamp examination, direct ophthalmoscopy, and non-cycloplegic refraction. In this study, both eyes were measured. All participants underwent measurement of uncorrected distance visual acuity (UCDVA) at 5 m using a standard logarithmic visual acuity E chart, with results recorded in logMAR scores. Visual acuity was tested with and without refractive correction for participants wearing spectacles. Previous studies have shown that, in children over 10 years old, non-cycloplegic refraction measurements are as accurate as cycloplegic measurements (14). In this study, refraction was assessed using non-cycloplegic measurement with an automatic computerized refractometer (-HRK-7000A, Huvitz Co. Ltd.). Each student’s eyes were measured thrice in a darkened room. Additional examinations were conducted if the difference in refractive error (RE) between readings was 0.50 diopters (D) or greater. The mean values of spherical diameter (SD) and cylindrical diameter (CD) were used for analysis.

Uncorrected visual acuity (UCVA) ≤ 0 logMAR was considered normal vision. The spherical equivalent refraction (SER) was converted by adding the spherical refraction and half of the cylindrical refraction. Myopia was defined as UCVA > 0 logMAR in either eye combined with an SER < = − 0.50 D1. Myopia severity was classified into three levels according to SER: mild myopia, −3.0 D < SER < −0.5 D; moderate myopia, −6.0 D < SER ≤ −3.0 D; and severe myopia, SER ≤ −6.00D1.

### Age at menarche

In this study, the age at menarche was self-reported by students. Based on their age at menarche, the students were arbitrarily divided into three groups to balance the sample size across groups: those who experienced menarche before 12 years of age were placed in the early menarche group, those at age 12 were placed in the normal menarche group, and those older than 12 years were placed in the late menarche group.

### Confounders

Physical examination: Physicians from the Department of Health Medical Center conducted other physical examinations, including measurements of height (meters), weight (kilograms), heart rate (times per min), and blood pressure (BP; mmHg). Body mass index (BMI) was calculated as body weight (kg)/height (m)2.

Pulmonary function test (PFT): Pulmonary function equipment (COSMED) was used to measure the students’ pulmonary function. The following measurements were recorded and used for analysis: forced vital capacity (FVC), forced expiratory volume in 1 s (FEV1), FVC% (the percentage of the actual FVC compared to the predicted value based on age, gender, and height), and FEV1% (the percentage of the actual FEV1 compared to the predicted value based on age, gender, and height).

Other variables: A structured questionnaire was administered through face-to-face interviews by a trained doctor to collect information about demographics, sociological background, the vision conditions of parents, eye-related behaviors, and the school to which each student was admitted. To avoid interviewer bias, a fixed team administered all questionnaires.

### Quality control

Before the study started, all research team members, including three experienced ophthalmologists, two qualified optometrists, and one postgraduate student, were trained. All instruments were checked and adjusted before the examination.

### Data analysis

Data were summarized as proportions for categorical variables and as mean± standard deviation (SD) for continuous variables. Variables were compared among the three menarche groups using chi-squared tests and analysis of variance (ANOVA), and the association between age at menarche and myopia was analyzed using univariate and multivariate logistic regression. A two-sided *p*-value of < 0.05 was considered statistically significant. All statistical analyses were conducted using SPSS 19.0.

## Results

### General characteristics of participants classified by age at menarche

Among the 376 participants with menarche, 115 girls were classified into the early menarche group, 185 into the normal menarche group, and 76 into the late menarche group. Early menarche was associated with higher weight and BMI. The other characteristics of the 376 enrolled students, according to age at menarche, are summarized in Table 1. There were no significant differences between the three groups in terms of age, height, myopic condition of parents, academic performance (based on the prestige of the schools they were admitted to), heart rate, BP, and PFT indexes.

**Table 1:** Characteristics of the study population according to age at menarche.

Variables	Early menarche	Normal menarche	Late menarche	*p*
	*N* = 115	*N* = 185	*N* = 76	
Age (year)	15.0 ± 0.5	15.0 ± 0.5	15.0 ± 0.2	0.423
Height (cm)	161.3 ± 5.8	161.3 ± 5.7	161.8 ± 5.0	0.186
Weight (Kg)	55.9 ± 11.4	53.6 ± 8.8	51.2 ± 8.4	0.005
BMI (Kg/m^2^)	21.4 ± 4.1	20.6 ± 3.0	19.6 ± 2.9	0.001
Parental myopia				
Father (n,%)	40 (34.8)	71 (38.4)	27 (35.5)	0.798
Mother (n,%)	49 (42.6)	74 (38.4)	28 (36.8)	0.675
Father and mother (n,%)	25 (21.7)	45 (24.3)	18 (23.7)	0.878
Academic level				
Low (n,%)	30 (26.1)	42 (22.7)	25 (32.9)	0.461
Middle (n,%)	62 (53.9)	97 (52.4)	36 (47.4)	
High (n,%)	23 (20.0)	46 (24.9)	15 (19.7)	
Heart rate (time/min)	77.1 ± 13.0	75.0 ± 12.7	76.0 ± 12.6	0.325
Blood pressure				
SBP (mmHg)	109.3 ± 13.1	108.6 ± 11.8	106.0 ± 11.3	0.161
DBP (mmHg)	66.5 ± 9.4	65.4 ± 7.4	65.5 ± 7.3	0.517
PET				
FVC (liter)	2.95 ± 0.41	2.87 ± 0.39	2.88 ± 0.40	0.268
FVC%	93.1 ± 11.6	92.3 ± 17.3	91.2 ± 12.8	0.747
FEV1 (liter)	2.69 ± 0.48	2.61 ± 0.50	2.61 ± 0.35	0.371
FEV1%	92.2 ± 12.3	92.1 ± 23.6	90.6 ± 11.2	0.838
Hyperopia				
Left eye	1 (0.9)	2 (1.1)	0 (0.0)	0.668
Right eye	0 (0.0)	3 (1.6)	0 (0.0)	0.210

Early menarche: menarche age<12; normal menarche: menarche age = 12; and late menarche: menarche age>12. PFT, pulmonary function test; FVC, forced vital capacity; FEV1, forced expiratory volume in 1 s; FVC%, the percentage of the actual FVC compared to the predicted FVC; FEV1%, the percentage of the actual FEV1 compared to the predicted FEV1.

### Ocular parameters by age at menarche

Table 2 demonstrates that the girls in the early menarche group had the lowest values of uncorrected visual acuity (logMAR), spherical diameter (SD), and spherical equivalent refraction (SER) in both eyes. However, cylindrical diameter (CD) values were not significantly different among the three groups.

**Table 2:** Ocular parameters of the participants by age at menarche.

Variables	Early menarche	Normal menarche	Late menarche	*p*
	*N* = 115	*N* = 185	*N* = 76	
Right eye				
LogMAR	−0.63 ± 0.34	−0.57 ± 0.34	−0.45 ± 0.37	0.003
SD	−2.93 ± 1.76	−2.60 ± 1.95	−2.16 ± 1.88	0.025
CD	−0.82 ± 0.69	−0.74 ± 0.70	−0.62 ± 0.53	0.138
SER	−3.30 ± 1.82	−2.91 ± 2.09	−2.43 ± 1.96	0.013
Left eye				
LogMAR	−0.63 ± 0.39	−0.51 ± 0.37	0.39 ± 0.39	0.000
SD	−2.64 ± 1.91	−1.98 ± 2.23	−1.48 ± 2.08	0.001
CD	−1.01 ± 0.72	−1.00 ± 0.79	−0.82 ± 0.64	0.178
SER	−3.12 ± 2.02	−2.41 ± 2.32	−1.87 ± 2.21	0.001

Early menarche: menarche age<12; normal menarche: menarche age = 12; and late menarche: menarche age > = 13. SD, spherical diameter; CD, cylindrical diameter; SER, spherical equivalent refraction.

### Myopia prevalence by age at menarche

The results in Table 3 demonstrate that the prevalence of myopia in the right eye was highest in the early menarche group (95.7%), followed by the normal menarche group (87.6%) and the late menarche group (86.6%). A similar pattern was observed in the left eye, with myopia prevalence of 89.6, 77.8, and 68.4% in the early, normal, and late menarche groups, respectively. The prevalence of myopia differed significantly across the menarche age groups in both eyes (*p* = 0.048 for the right eye, *p* = 0.001 for the left eye). Among the girls who experienced early menarche, moderate myopia was the most prevalent type (52.2% in the right eye and 44.3% in the left eye), while for girls with normal or late menarche, mild myopia was most prevalent.

**Table 3:** Comparison of myopia prevalence between the different menarche age groups using left and right eye data.

Variables	Early menarche	Normal menarche	Late menarche	*p*
	*N* = 115	*N* = 185	*N* = 76	
Right eye				
Myopia				0.048
No	5 (4.3)	23 (12.4)	10 (13.2)	
Yes	110 (95.7)	162 (87.6)	66 (86.6)	
Myopia severity				0.014
No	5 (4.3)	23 (12.4)	10 (13.2)	
Mild	44 (38.3)	78 (42.2)	38 (50.0)	
Moderate	60 (52.2)	66 (35.7)	24 (31.6)	
Severe	6 (5.2)	18 (9.7)	4 (5.3)	
Left eye				
Myopia				0.001
No	12 (10.4)	41 (22.2)	24 (31.6)	
Yes	103 (89.6)	144 (77.8)	52 (68.4)	
Myopia severity				0.002
No	12 (10.4)	41 (22.2)	24 (31.6)	
Mild	43 (37.4)	69 (37.3)	31 (40.8)	
Moderate	51 (44.3)	59 (31.9)	14 (18.4)	
Severe	9 (7.8)	16 (8.6)	7 (9.2)	

Early menarche: menarche age <12 years old; normal menarche: menarche age = 12 years old; and late menarche: menarche age >12 years old.

### Multivariate regression analysis of myopia and age at menarche

Table 4 shows that among the girls aged 15, age at menarche was negatively associated with the risk of myopia in both eyes, with a stronger association observed in the left eye (*p* < 0.001). After adjusting for all available confounders, the relationship between age at menarche and the risk of myopia in the right eye became marginally significant (*p* = 0.066), while the association remained significant in the left eye (*p* = 0.001). The girls with early menarche were more likely to develop myopia compared to those with normal or late menarche.

**Table 4:** Multivariate logistic regression analysis of myopia and age at menarche using left and right eye data.

Models	*β*	SE	*p*	R2	AIC	VIF
Right eye						
Model 1	−0.601	0.277	0.030	0.016	245.5226	1.000
Model 2	−0.538	0.282	0.044	0.029	248.9927	1.040
Model 3	−0.587	0.289	0.042	0.049	207.0471	1.043
Model 4	−0.561	0.305	0.066	0.095	196.7198	1.047
Left eye						
Model 1	−0.793	0.218	<0.001	0.045	372.0574	1.000
Model 2	−0.742	0.223	0.001	0.066	370.3294	1.040
Model 3	−0.764	0.225	0.001	0.078	296.309	1.043
Model 4	−0.796	0.238	0.001	0.127	284.8137	1.047

Early, normal, and late menarche were coded as 1,2, and 3, respectively (early menarche: menarche age <12; normal menarche: menarche age = 12; and late menarche: menarche age >12). AIC, Akaike Information Criterion; VIF, Variance Inflation Factor. Model 1: Unadjusted. Model 2: Adjusted for BMI, heart rate, SBP, and DBP. Model 3: Model 2 plus adjustments for FVC, FVC%, FEV1, and FEV1%. Model 4: Model 3 plus adjustments for parental myopia, father’ and mother’, and academic performance.

### Multivariate regression analysis of moderate/severe myopia and age at menarche

When considering the severity of myopia, we performed a multivariate logistic regression analysis to examine the relationship between age at menarche and the risk of moderate/severe myopia. Table 5 shows that age at menarche was negatively and significantly associated with the risk of moderate/severe myopia in both eyes (*p* < 0.05), even after adjusting for confounders. The girls aged 15 with early menarche had a significantly higher risk of mild/severe myopia compared to their peers with normal or late menarche.

**Table 5:** Multivariate logistic regression analysis of moderate/severe myopia and age at menarche using right and left eye data.

Models	*β*	SE	*p*	R2	AIC	VIF
Right eye						
Model1	−0.374	0.166	0.024	0.017	515.9249	1.000
Model2	−0.359	0.171	0.036	0.036	516.6783	1.040
Model3	−0.365	0.172	0.033	0.045	431.1128	1.043
Model4	−0.363	0.176	0.039	0.086	423.77	1.047
Left eye						
Model1	−0.615	0.174	<0.001	0.042	502.6674	1.000
Model2	−0.605	0.178	0.001	0.055	506.4649	1.040
Model3	−0.614	0.179	0.001	0.060	419.7281	1.043
Model4	−0.616	0.182	0.001	0.098	413.3949	1.047

Early, normal, and late menarche were coded as 1, 2, and 3, respectively (early menarche: menarche age <12; normal menarche: menarche age = 12; and late menarche: menarche age >12). AIC, Akaike Information Criterion; VIF, Variance Inflation Factor. Model 1: Unadjusted. Model 2: Adjusted for BMI, heart rate, SBP, and DBP. Model 3: Model 2 plus adjustments for FVC, FVC%, FEV1, and FEV1%. Model 4: Model 3 plus adjustments for parental myopia, father’ and mother’, and academic performance.

## Discussion

This study demonstrated that girls who experienced early menarche had lower values of ocular indexes, such as visual acuity, spherical diameter, cylindrical diameter, and spherical equivalent refraction, and a higher risk of myopia, especially moderate/severe myopia, than those with normal or late menarche at the age of 15.

These results are consistent with studies that have shown a negative relationship between age at menarche onset and myopia in female adults (9, 10). However, our results differ from other studies on teenagers. According to a series of national surveys of Chinese adolescents, post-menarche girls had a higher prevalence of myopia than pre-menarche girls at ages 10–14 years, but not at the age of 15 (15). This may be because by age 15, nearly all girls had their menarche and are therefore classified as post-menarche, making it difficult to detect the influence of menarche at this age. In another cohort study conducted in Singapore, in which school girls aged 7–10 years were included and followed up until the age of 14, the results showed that the onset age of puberty, instead of menarche, was associated with the age of myopia onset (12).

In this study, we further confirmed the negative association between age at menarche and the risk of myopia in 15-year-old schoolgirls, which is older than the age examined in other studies. Therefore, the exposure time to sex hormones such as estrogen, progestin, and lutein in girls with earlier menarche is much longer than those in other studies. Regarding the role of sex hormones in the eyes, a 2-year follow-up cohort study by Wang et al. (15) showed that increased estradiol was associated with more negative refractive error and axial length elongation in Chinese children (16). Similarly, Xie also reported significant differences in the levels of estradiol, testosterone, follicle-stimulating hormone, and lutein hormone between myopia and non-myopia groups (17). Estrogen receptors have been found in multiple parts of the human eyes, including the retinal pigment epithelium (RPE) (18), sclera (19), cornea (20), and aqueous fluid in the vitreous body (19). The molecular mechanisms related to sex hormones include estrogen-regulating matrix metalloproteinases (MMPs) in the RPE and sclera (21, 22), which may contribute to the remodeling of the scleral and cornea. Insulin-like growth factor-1 (IGF-1) may be involved in myopia (23, 24). by contributing to ocular axial elongation (25, 26).

After adjusting for all possible confounders, including pulmonary function, which may be positively related to physical activity, as noted by Ji J (23), the relationship between age at menarche and myopia remained significant in both eyes. This suggests that early menarche is an independent risk factor for myopia in teenagers aged 15 years.

In our study, we also found that ocular measurements such as SD and SER and the proportions of myopia were more adverse in the right eye than in the left eye in each menarche age group. A potential explanation may involve ocular dominance and asymmetry in visual behavior, which expose the right eye to more risk factors. Overexposure to other risk factors may reduce the influence of age at menarche on myopia, which is consistent with the regression analysis results showing that the regression coefficients were slightly smaller in the right eye than in the left eye.

As early menarche or puberty is an independent risk factor of myopia, especially for the moderate to severe degree of myopia, evidence-based interventions should be emphasized when signs of early puberty, such as early menarche, are observed. These interventions include increasing outdoor activities (recommended at least 2 h per day) (24), cultivating the habit of taking breaks during eye use (27), undergoing annual ocular examinations (28), and the proper use of low-dose atropine and optical interventions, such as orthokeratology or defocus glasses (29).

### Strengths and limitations

This study has several strengths. The participants had a high homogeneity of age and shared similar living and educational environments, which helped reduce potential confounding factors such as age in the analysis of myopia. The measurement of SER is more accurate for identifying myopia than regular vision acuity tests or questionnaires, which have been used in other large-scale studies. Furthermore, we surveyed the participants through face-to-face interviews, which may provide more accurate information about age at menarche than a self-administrated questionnaire. Finally, this study is the first to include both spherical and cylindrical lens measurements and to investigate the incidence and severity of myopia in the right and left eye separately to study the relationship between myopia and menarche.

However, several limitations should also be noted. First, the sample size was relatively small and drawn from a single site, making selection bias inevitable and limiting the generalizability of the results to all teenage girls. Second, as this was a cross-sectional study, we could not establish a causal relationship between menarche and myopia, although it is unlikely that myopia influences the timing of menarche. Third, we had no information about hormone levels, the gold standard for measuring puberty. Therefore, the underlying mechanism linking menarche to myopia is still unclear. Fourth, we did not measure the axial lengths of these participants, which can more accurately and completely reflect the degree of myopia. Finally, the age at menarche was self-reported, which may have introduced recall bias. In addition, data on lifestyle behaviors, such as eye usage habits, outdoor activity, screen time, and sleep quality, were unavailable for analysis, which may cause bias in the results.

## Conclusion

Early menarche, as an independent risk factor, can increase the risk of myopia in adolescents. More active interventions should be considered to reduce the risk of myopia in children with early menarche. In addition, longitudinal studies on the increased risk of myopia associated with early onset of puberty are warranted.

## Data Availability

The raw data supporting the conclusions of this article will be made available by the authors, without undue reservation.

## References

[1] Morgan IG Ohno-Matsui K Saw SM. Myopia. Lancet. (2012) 379:1739–48. doi: 10.1016/S0140-6736(12)60272-4 22559900

[2] Rudnicka AR Kapetanakis VV Wathern AK Logan NS Gilmartin B Whincup PH. Global variations and time trends in the prevalence of childhood myopia, a systematic review and quantitative meta-analysis: implications for aetiology and early prevention. Br J Ophthalmol. (2016) 100:882–90. doi: 10.1136/bjophthalmol-2015-307724, PMID: 26802174 PMC4941141

[3] Ikuno Y. Overview of the complications of high myopia. Retina. (2017) 37:2347–51. doi: 10.1097/IAE.0000000000001489, PMID: 28590964

[4] Fricke TR Holden BA Wilson DA Schlenther G Naidoo KS Resnikoff S. Global cost of correcting vision impairment from uncorrected refractive error. Bull World Health Organ. (2012) 90:728–38. doi: 10.2471/BLT.12.104034, PMID: 23109740 PMC3471057

[5] Wu PC Huang HM Yu HJ Fang PC Chen CT. Epidemiology of myopia. Asia Pac J Ophthalmol (Phila). (2016) 5:386–93. doi: 10.1097/APO.0000000000000236, PMID: 27898441

[6] Andersen SW Trentham-Dietz A Gangnon RE. Breast cancer susceptibility loci in association with age at menarche, age at natural menopause and the reproductive lifespan. Cancer Epidemiol. (2014) 38:62–5. doi: 10.1016/j.canep.2013.12.001, PMID: 24373701 PMC4023814

[7] Kwan BS Yang J Jo HC Baek JC Kim RB Park JE. Age at menarche and its association with adult-onset metabolic syndrome and related disorders in women: a cross-sectional study of a nationally representative sample over 10 years. Asia-Pac J Public Health. (2024) 36:558–64. doi: 10.1177/10105395241271174, PMID: 39126335

[8] Mishra SR Chung HF Waller M Mishra GD. Duration of estrogen exposure during reproductive years, age at menarche and age at menopause, and risk of cardiovascular disease events, all-cause and cardiovascular mortality: a systematic review and meta-analysis. BJOG. (2021) 128:809–21. doi: 10.1111/1471-0528.16524, PMID: 32965759

[9] Lyu IJ Kim MH Baek SY Kim J Park KA Oh SY. The association between menarche and myopia: findings from the Korean National Health and nutrition examination, 2008–2012. Invest Ophthalmol Vis Sci. (2015) 56:4712–8. doi: 10.1167/iovs.14-16262, PMID: 26207307

[10] Lyu IJ Oh SY. Association between age at menarche and risk of myopia in the United States: NHANES 1999–2008. PLoS One. (2023) 18:e0285359. doi: 10.1371/journal.pone.0285359 37146094 PMC10162551

[11] Xu R Jan C Song Y Dong Y Hu P Ma J. The association between menarche and myopia and its interaction with related risk behaviors among Chinese school-aged girls: a nationwide cross-sectional study. J Dev Orig Health Dis. (2020) 11:573–9. doi: 10.1017/S204017442000077X, PMID: 32799955

[12] Yip VCH Pan CW Lin XY Lee YS Gazzard G Wong TY. The relationship between growth spurts and myopia in Singapore children. Invest Ophthalmol Vis Sci. (2012) 53:7961–6. doi: 10.1167/iovs.12-10402, PMID: 23150611

[13] Ma N Shi D Dang JJ Zhong PL Liu YF Cai S. Secular trends and urban-rural disparities in the median age at menarche among Chinese han girls from 1985 to 2019. World J Pediatr. (2023) 19:1162–8. doi: 10.1007/s12519-023-00723-9, PMID: 37093553

[14] Hashemi H Khabazkhoob M Asharlous A Soroush S Yekta AA Dadbin N. Cycloplegic autorefraction versus subjective refraction: the Tehran eye study. Br J Ophthalmol. (2016) 100:1122–7. doi: 10.1136/bjophthalmol-2015-307871, PMID: 26541436

[15] Xu R Zhong P Jan C Song Y Xiong X Luo D. Sex disparity in myopia explained by puberty among Chinese adolescents from 1995 to 2014: a Nationwide cross-sectional study. Front Public Health. (2022) 10:833960. doi: 10.3389/fpubh.2022.833960, PMID: 35712300 PMC9196902

[16] Wang J Cheng T Zhang B Xiong S Zhao H Li Q. Puberty could regulate the effects of outdoor time on refractive development in Chinese children and adolescents. Br J Ophthalmol. (2021) 105:191–7. doi: 10.1136/bjophthalmol-2019-315636 32299828 PMC7848068

[17] Xie H Mao X Yang H Xie Z Pan Y Gao Y. Analysis on the relationship between adolescent myopia and serum sex hormone. Zhonghua Yi Xue Za Zhi. (2014) 94:1294–7. doi: 10.3760/cma.j.issn.0376-2491.2014.17.004 PMID: 25142847

[18] Marin-Castaño ME Elliot SJ Potier M. Regulation of estrogen receptors and MMP-2 expression by estrogens in human retinal pigment epithelium. Invest Ophthalmol Vis Sci. (2003) 44:50–9. doi: 10.1167/iovs.01-1276, PMID: 12506055

[19] Gupta PD Johar K Nagpal K Vasavada AR. Sex hormone receptors in the human eye. Surv Ophthalmol. (2005) 50:274–84. doi: 10.1016/j.survophthal.2005.02.005, PMID: 15850816

[20] Aydin E Demir HD Demirturk F Caliskan AC Aytan H Erkorkmaz U. Corneal topographic changes in premenopausal and postmenopausal women. BMC Ophthalmol. (2007) 7:9. doi: 10.1186/1471-2415-7-9, PMID: 17501998 PMC1877796

[21] Guggenheim JA McBrien NA. Form-deprivation myopia induces activation of scleral matrix metalloproteinase-2 in tree shrew. Invest Ophthalmol Vis Sci. (1996) 37:1380–95. 8641841

[22] Nickla DL Damyanova P Lytle G. Inhibiting the neuronal isoform of nitric oxide synthase has similar effects on the compensatory choroidal and axial responses to myopic defocus in chicks as does the non-specific inhibitor L-NAME. Exp Eye Res. (2009) 88:1092–9. doi: 10.1016/j.exer.2009.01.012, PMID: 19450449 PMC2834415

[23] Ji J Wang S Jian LY. Physical activity and lung function growth in a cohort of Chinese school children: a prospective study. PLoS One. (2013) 8:e66098. doi: 10.1371/journal.pone.0066098, PMID: 23840406 PMC3686802

[24] Saxena R Dhiman R Gupta V Phuljhele S Mahajan A Rakheja V. Prevention and management of childhood progressive myopia: national consensus guidelines. Indian J Ophthalmol. (2023) 71:2873–81. doi: 10.4103/IJO.IJO_387_23, PMID: 37417137 PMC10491088

[25] Penha AM Schaeffel F Feldkaemper M. Insulin, insulin-like growth factor-1, insulin receptor, and insulin-like growth factor-1 receptor expression in the chick eye and their regulation with imposed myopic or hyperopic defocus. Mol Vis. (2011) 17:1436–48. PMID: 21655358 PMC3108898

[26] Feldkaemper MP Neacsu I Schaeffel F. Insulin acts as a powerful stimulator of axial myopia in chicks. Invest Ophthalmol Vis Sci. (2009) 50:13–23. doi: 10.1167/iovs.08-1702, PMID: 18599564

[27] Li R Zhang J Zhang Y Tang W Ao D He L. Lifestyle and risk of developing myopia in school children in Chongqing. China Front Med. (2024) 11:1439833. doi: 10.3389/fmed.2024.1439833, PMID: 39444822 PMC11497096

[28] Wang W Xiang Y Zhu L Zheng S Ji Y Lv B. Myopia progression and associated factors of refractive status in children and adolescents in Tibet and Chongqing during the COVID-19 pandemic. Front Public Health. (2022) 10:993728. doi: 10.3389/fpubh.2022.993728, PMID: 36324441 PMC9619363

[29] Lee SH Tsai PC Chiu YC Wang JH Chiu CJ. Impact of orthokeratology and low-dose atropine on corneal biomechanics and myopia progression in children. Ophthalmic Physiol Opt. (2025) 45:565–76. doi: 10.1111/opo.13446, PMID: 39803986

